# FAF1 downregulation by *Toxoplasma gondii* enables host IRF3 mobilization and promotes parasite growth

**DOI:** 10.1111/jcmm.16889

**Published:** 2021-08-31

**Authors:** Fei‐Fei Gao, Juan‐Hua Quan, In‐Wook Choi, Yeon‐Jae Lee, Seul‐Gi Jang, Jae‐Min Yuk, Young‐Ha Lee, Guang‐Ho Cha

**Affiliations:** ^1^ Brain Korea 21 FOUR Project for Medical Science Chungnam National University Daejeon Korea; ^2^ Department of Medical Science and Department of Infection Biology Chungnam National University Daejeon Korea; ^3^ Department of Gastroenterology Affiliated Hospital of Guangdong Medical University Zhanjiang China

**Keywords:** AKT, ARPE‐19 cells, FAF1, IRF3, *Toxoplasma gondii*

## Abstract

Fas‐associated factor 1 (FAF1) has gained a reputation as a member of the FAS death‐inducing signalling complex. However, the role of FAF1 in the immunity response is not fully understood. Here, we report that, in the human retinal pigment epithelial (RPE) cell line ARPE‐19 cells, FAF1 expression level was downregulated by *Toxoplasma gondii* infection, and PI3K/AKT inhibitors reversed *T*.* gondii*‐induced FAF1 downregulation. In silico analysis for the FAF1 promoter sequence showed the presence of a FOXO response element (FRE), which is a conserved binding site for FOXO1 transcription factor. In accordance with the finding, FOXO1 overexpression potentiated, whereas FOXO1 depletion inhibited intracellular FAF1 expression level. We also found that FAF1 downregulation by *T*.* gondii* is correlated with enhanced IRF3 transcription activity. Inhibition of PI3K/AKT pathway with specific inhibitors had no effect on the level of *T*.* gondii*‐induced IRF3 phosphorylation but blocked IRF3 nuclear import and ISGs transcription. These results suggest that *T*.* gondii* can downregulate host FAF1 in PI3K/AKT/FOXO1‐dependent manner, and the event is essential for IRF3 nuclear translocation to active the transcription of ISGs and thereby *T*.* gondii* proliferation.

## 1 INTRODUCTION

*Toxoplasma gondii* is a ubiquitous, intracellular, apicomplexan parasite of warm‐blooded animals and is one of the most common parasite of human and other animals, which affects approximately one third of the global human population.^1^ *Toxoplasma gondii* plays a crucial role in retinochoroiditis in immunocompetent and immunosuppressed individuals, and ocular lesions may be present in up to 20% of infected patients.^2^ The retinal pigment epithelium (RPE) cell is an integral part of the neuroretina in the posterior segment of the eye, whose normal functions are for light absorption, epithelial conveyance, spatial ion buffering, visual cycle, phagocytosis, secretion, elimination of oxidized or damaged materials and immune modulation. Various molecular responses of human RPE cells to infection with *T*.* gondii* has been presented by several research groups.^3^, ^4^, ^5^, ^6^, ^7^ However, the host TBK1/IRF3 signalling for *T*.* gondii* infection and growth in ARPE‐19 cells is elusive.

Interferon regulatory factor 3 (IRF3), key transcriptional mediator of type I interferon (IFN)‐dependent immune responses, plays an essential role in the innate immune response against DNA and RNA viruses.^8^, ^9^ IRF3 is constitutively expressed in several tissues and localized in the cytoplasm in an unstimulated condition. During the course of infection, single‐ or double‐stranded infectious RNAs from virus accumulated inside cells are recognized by PRRs, which recruit the kinase TANK‐binding kinase 1 (TBK1) and active IRF3 by phosphorylating its C‐terminal region.^10^, ^11^, ^12^ The activation leads to IRF3 dimerization and translocation from cytosol to nucleus and binds to a specific promoter sequence, which conserved enhancer motifs named ISREs to induce transcription of ISGs, which would contribute to various immune responses. Interestingly, it has also been reported that instead of detrimental effect to parasite, TBK1/IRF3 activity promotes *T*.* gondii's* efficient proliferation.^13^, ^14^

In addition to TBK1/IRF3 pathway, several reports have demonstrated that PI3K is also involved in the *T*.* gondii* infection event through activation of AKT.^15^, ^16^ In response to insulin or growth factors, AKT directly regulates the phosphorylation of FOXO transcription factors, which cause the export of FOXO from the nucleus to the cytoplasm, and thereby resulted in inhibition of FOXOs transcription activity and downstream gene expression.^17^ Although this signalling pathway has been intensively studied in the view of metabolic syndromes, it also provided issues to other fields, especially in infection biology. There were challenges to reveal the role of FOXO transcription factors in the immunobiology^18^, ^19^, ^20^; for example, FOXO3a was reported to suppress IRF7 transcription and negatively mediate innate immune response.^21^

Given the important role of PI3K/AKT pathways in the regulation of metabolic processes and in view of emerging information regarding the *T*.* gondii*‐related immunological and pathological activities of FOXO transcription factors, and our observation of PI3K/AKT‐dependent IRF3 activity by *T*.* gondii*, we established the hypothesis that FAF1 is an essential link between *T*.* gondii*‐induced TBK1 and PI3K signalling pathways for *T*.* gondii* growth.

Fas‐associated factor (FAF1), initially identified as a Fas‐binding protein that potentiates Fas‐induced apoptosis, participates in diverse mechanisms that promote cell death and thus is thought to act as a tumour suppressor.^22^, ^23^ In addition to its inhibitory action on tumorigenesis, FAF1 is also involved in diverse biological processes. It has also been demonstrated that FAF1 plays critical roles in defence against pathogens infection, and as such, FAF1 can protect the host form Listeria infection by positively regulating NADPH oxidase‐mediated ROS production,^24^ and FAF1 also plays a novel role in negatively regulating virus‐induced IFN‐β production and the antiviral response by inhibiting the translocation of active, phosphorylated IRF3 from the cytosol to the nucleus.^25^ However, FAF1's role in protozoan infection is not fully understood yet.

In this study, we provide evidences that *T*.* gondii* requires activation of host TBK1/IRF3 and downstream genes (ISGs) expression for its efficient infection and growth in ARPE‐19 cells. In addition, we also revealed that *T*.* gondi*i controls the expression of host FAF1 in a PI3K/AKT‐dependent manner. A property of FAF1 as a “trapper” for IRF3 nuclear import negatively contributes to the required *T*.* gondii*‐induced ISGs expression, and indeed, impairment of FAF1 expression resulted in enhanced transcription of host ISGs. Combined together, our results identified a regulatory link between the TBK1/IRF3 pathway and PI3K/AKT pathway, which is controlled by *T*.* gondii* infection and essential for its growth. The present study will provide the insights about how *T*.* gondii* modulates the host cell signalling pathway for its growth and about the immunopathological basis of ocular toxoplasmosis.

## 2 MATERIALS AND METHODS

### 2.1 Antibodies and reagents

The following antibodies were used: rabbit antibodies specific for IRF3, phospho‐IRF3 (Ser396), TBK1 and phospho‐TBK1 (Ser172) were purchased from Cell Signaling Technology Inc. Anti‐α‐Tubulin antibody was obtained from Santa Cruz Biotechnology and anti‐fibrillarin antibody was from Abcam. The following secondary antibodies, anti‐rabbit‐horseradish peroxidase (HRP) and anti‐mouse HRP were from Jackson Immuno Research Laboratories. Goat anti‐Rabbit IgG (H+L) Highly Cross‐Adsorbed Secondary Antibody‐Alexa Fluor 488, Alexa Fluor 647 and Goat anti‐Mouse IgG (H+L) Highly Cross‐Adsorbed Secondary Antibody‐Alexa Fluor 488, Alexa Fluor 568 were purchased from Thermo Fisher Scientific.

PI3K‐specific inhibitors, Pictilisib (GDC‐0941) and ZSTK474, were obtained from Selleck Chemicals. AKT inhibitor, Akti1/2, was got from Sigma.

### 2.2 Cell culture

The human retinal pigment epithelial cell line ARPE‐19, which consists of highly polarized cells that from the outer blood–retina barrier between the photoreceptors of the neurosensory retina and vascularized choroid, was obtained from the American Type Culture Collection (ATCC). Cells were routinely cultured under 5% CO_2_ at 37°C in DMEM/F12 medium (WelGENE) supplemented with 10% heat‐inactivated foetal bovine serum (FBS, Gibco BRL) and 1% antibiotic‐antimycotic reagents (Gibco BRL). The cells were passaged with 0.25% Trypsin‐EDTA (Life Technologies) every 3–4 days. ARPE‐19 cells between passages 4 and 8 were used in this study.

### 2.3 Parasites

*Toxoplasma gondii* tachyzoites RH strain was maintained in ARPE‐19 cells at 37°C, 5% CO_2_ and passaged for every 2–3 days. RH expressing transgenic green fluorescent protein (GFP‐RH) and red fluorescent protein (RFP‐RH) was kindly provided by Dr. Yoshifumi Nishikawa (Obihiro University of Agriculture and Veterinary Medicine) and incubated same condition with RH.

### 2.4 Preparation of *T*.* gondii* ESA

Excretory/secretory antigen (ESA) from *T*.* gondii* was produced as described previously,^26^ with slight modifications. Briefly, freshly purified tachyzoites (1 × 10^8^) from the peritoneal cavity were incubated at 37°C for 3 h under mild agitation in test tubes containing 1.0 ml Hank's balanced salt solution (HBSS) (Gibco BRL). After centrifugation for 5 min at 6000 *g*, the supernatant including ESA was saved. The protein concentration was measured by means of the Bio‐Rad DC protein assay method with BSA as the standard, and samples were stored at −70°C until been used.

### 2.5 Western blot analysis

SDS‐PAGE and Western blot analysis were performed to determine numerous proteins expression. ARPE‐19 cells were cultured in 60‐mm plates and underwent serum starved for 3 h to remove stimulations from serum factors. Cells were stimulated with poly (dA:dT), RH, ESA, siRNA or plasmid DNA as indicated. Proteins were extracted with PRO‐PREP Protein Extraction Solution (iNtRON Biotechnology) and then splitted for 30 min on ice followed by boiling for 5 min. The same amount of proteins was loaded into the SDS‐PAGE gel and separated by electrophoresis. Then proteins were transferred to polyvinylidene difluoride (PVDF) membrane (Bio‐Rad Laboratories). The membranes were blocked with 5% skim milk in Tris‐buffered saline including 0.1% Tween 20 (TBST) for 1 h at room temperature. After being washed twice in TBST for 5 min, membranes were incubated with indicated primary antibodies (1:1000 diluted in 5% BSA supplemented with TBST) overnight at 4°C. Following washed three times with TBST, membranes were incubated with HRP‐conjugated anti‐rabbit or anti‐mouse secondary antibody (1:2000 diluted in 5% skim milk supplemented with TBST) for 2 h at room temperature. After three times washing, the membranes were soaked with Immobilon Western Chemiluminescent HRP Substrate (Merck Millipore), and chemiluminescence was detected using a Fusion Solo System (Vilber Lourmat).

### 2.6 Targeted gene silencing

One day before transfection, ARPE‐19 cells were cultured in a 6‐well plate with 3 ml normal growth media. The medium was removed and 500 μl fresh DMEM/F12 was added to the plate. Diluting siRNA duplex in 250 μl fresh DMEM/F12 to make final concentration of 5–100 nm. Lipofectamine™ RNAiMAX (Life Technologies Corporation) was gently mixed before use and then diluted 5–250 μl media, incubated for 5 min at room temperature. The diluted siRNA duplex and diluted Lipofectamine™ RNAiMAX were combined and incubated for 20 min at room temperature. The mixture was added to each well containing cells. The final volume in each well is 1 ml. The plate was mixed gently by hand rocking back and forth. After incubating 6 h at 37°C, the media was changed to normal growth media and incubated 48 h at 37°C. Gene knockdown efficiency was assayed by Western blot or immunocytochemistry.

### 2.7 Overexpression of FOXO1 and ISG15

Overexpression of FOXO1 and ISG15 was performed by pEGFP‐N1‐FOXO1 and p3x‐FLAG‐CMV‐10‐ISG15 plasmid DNA transfection. We used the Lipofectamine™ LTX Reagent with PLUS™ Reagent (Thermo Fisher Scientific) according to the manufacturer's protocol. Briefly, ARPE‐19 cells were cultured in a 6‐well plate with 3 ml normal growth media to be 70%–80% confluent at transfection. We dilute Lipofectamine™ LTX Reagent in fresh DMEM/F12 medium, dilute DNA in DMEM/F12 medium and then add PLUS™ Reagent. We add diluted DNA to diluted Lipofectamine™ LTX Reagent (1:1 ratio) and incubate at room temperature for 5 min. Gene overexpression efficiency was assayed by Western blot or RT‐PCR.

### 2.8 Preparation of the subcellular fraction lysates

Cytoplasmic and nuclear fractions of cells were isolated following the manufacturer's instructions (Active Motif). Briefly, ARPE‐19 cells cultured in 100‐mm plates were washed with 5 ml ice‐cold PBS/Phosphatase inhibitors. Aspirate solution out and add 3 ml ice‐cold PBS/Phosphatase inhibitors, and remove cells from dish by gently scraping with cell scraper; centrifuge cell suspension in a centrifuge pre‐cold at 4°C for 5 min at 200 *g*; discard supernatant and gently re‐suspend cells in 500 μl 1× Hypotonic Buffer by pipetting up and down several times; transfer to a pre‐chilled microcentrifuge tube; allow cells to swell by incubating for 15 min on ice; add 25 μl detergent and vortex 10 s at the highest setting; centrifuge suspension for 30 s at 14,000 *g*. The supernatant is the cytoplasmic fraction. We re‐suspend the pellet in 50 μl complete lysis buffer by pipetting up and down and then incubate suspension for 30 min on ice on a rocking platform set at 150 rpm; vortex 30 s at the highest setting; and centrifuge for 10 min at 14,000 *g* in a microcentrifuge pre‐cooled at 4°C. The supernatant is the nucleus fraction. We aliquot and store at −80°C, avoid freeze/thaw cycles.

### 2.9 Translocation of IRF3

ARPE‐19 cells seeded in 24‐well plate with glass coverslips were infected with GFP‐RH or RFP‐RH at MOI5 for 24 h, and fixed with 4% paraformaldehyde overnight at 4°C. The cells were blocking with 1% BSA in PBS containing 0.1% Triton X‐100 (PBST) for 30 min at room temperature and then incubated with IRF3 primary antibody (1:200 diluted) for 2 h at room temperature. Following three times washing with PBST, cells were incubated with Alexa Fluor^®^ 647 or 488 goat anti‐rabbit IgG secondary antibody. After washing three times by PBST, cells were mounted with VECTASHIELD HardSet antifade mounting solution with DAPI (Vector Laboratories) to stain nucleus. The samples were imaged by using Leica confocal microscope (Leica TCS SP8).

### 2.10 RNA isolation and RT‐PCR

Total RNA from stimulated cells was isolated using the Trizol reagent (Invitrogen), and cDNA was generated using M‐MLV reverse transcriptase kit (Invitrogen Life Technologies) as described by the manufacturer. All PCR reactions were performed with a MyCycler (Bio‐Rad) for 25 cycles. The primers were designed using Primer 3 software. mRNA sequence of the selected genes was obtained from NCBI website. The primer sequences are summarized in Table 1. Amplified products were electrophoresed in a 1.5% agarose gel and visualized with ethidium bromide. Quantification of mRNA was performed using an imaging densitometer (Bio‐Rad Laboratories, Inc).

**TABLE 1:** Human and *Toxoplasma gondii* primers used to investigate their expressions by RT‐PCR and qRT‐PCR

Name	Forward 5′−3′	Reverse 5′−3′	Length (bp)
FOXO1	AACCTGTCCTACGCCGACCTCA	GCTCGGCTTCGGCTCTTAGCAA	326
FAF1	ATTGGGACTTAGTGGCAGCT	GCATTACAGGTCGAAACGCT	174
IFNβ	GCCTCAAGGACAGGATGAAC	AGCCAGGAGGTTCTCAACAA	176
ISG15	GAGAGGCAGCGAACTCATCT	AGGGACACCTGGAATTCGTT	125
ISG56	GCCTTGCTGAAGTGTGGAGGAA	ATCCAGGCGATAGGCAGAGATC	125
GAPDH	GTCTCCTCTGACTTCAACAGCG	ACCACCCTGTTGCTGTAGCCAA	131
SAG1	GCTGTAACATTGAGCTCCTTGATTCCTG	CCGGAACAGTACTGATTGTTGTCTTGAG	357

### 2.11 Real‐time quantitative revere transcription polymerase chain reaction (qRT‐PCR)

qRT‐PCR was performed using Power SYBR^®^ Green PCR Master Mix (Applied Biosystems). The primers used in this study are summarized in Table 1. All reactions were performed with an ABI 7500 Fast Real‐Time PCR system (Applied Biosystems) under the following conditions: 95°C for 10 min, followed by 40 cycles of 95°C for 15 s and 60°C for 1 min. Relative gene expression levels were quantified based on the cycle threshold (Ct) values and normalized to the reference gene GAPDH. Each sample was measured in triplicate, and the gene expression levels were calculated using the 2^–ΔΔCt^ method.

### 2.12 Statistical analysis

All these experiments were repeated at least three times, independently, and the results were expressed as the mean ± standard deviation (SD). *p* values between groups were determined by a two‐tailed paired Student's *t* tests. *p* < 0.05 was considered as “statistically significant.”

## 3 RESULTS

### 3.1 *T*.* gondii* growth requires activation of host TBK1/IRF3 signalling pathway

**FIGURE 1:**
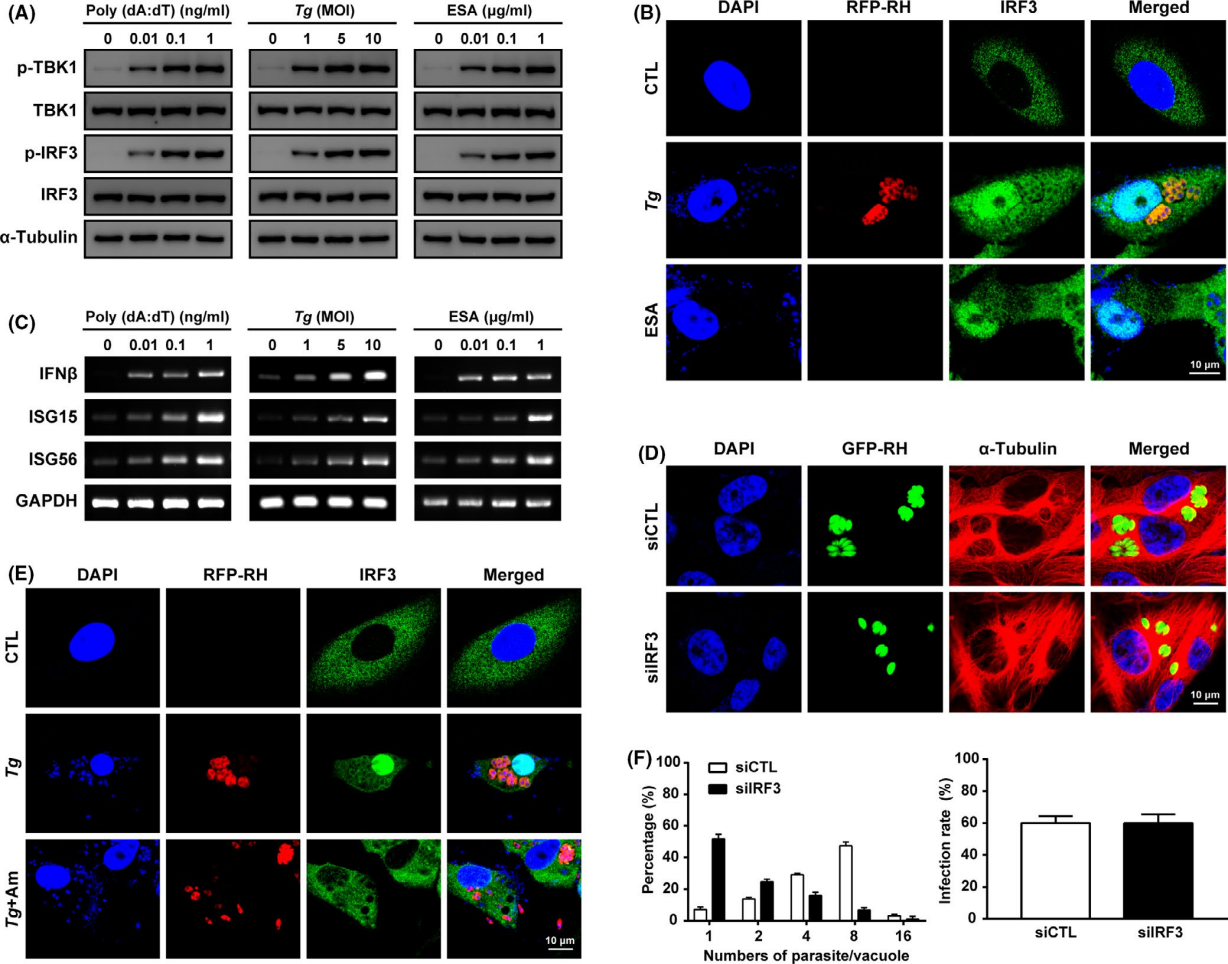
*Toxoplasma gondii* growth requires activation of host TBK1/IRF3 pathway. (A) Western blot analysis of phosphor‐TBK1/IRF3 and total TBK1/IRF3 in ARPE‐19 cells stimulated with poly (dA:dT) or *T*.* gondii* (*Tg*) or ESA. (B) ARPE‐19 cells were stimulated with *T*.* gondii* at MOI5 (*Tg*) or 1 μg/ml ESA (ESA) or unstimulated (CTL). IRF3 distribution was monitored by confocal fluorescence. (C) mRNA expression level of IRF3 target genes was measured by RT‐PCR. (D) ARPE‐19 cells cultured in 12‐well plates were infected by RFP‐RH at MOI5 for 24 h with TBK1 inhibitor Amlexanox (1 µM) pretreatment (*Tg* + Am) or not (*Tg)* or uninfected (CTL). The effect of TBK1 inhibitor on IRF3 translocation and *T*.* gondii* proliferation was measured by confocal fluorescence. (E) ARPE‐19 cells transiently transfected with IRF3 siRNA (siIRF3) or control scrambled siRNA (siCTL) were infected with GFP‐RH at MOI5 for 24 h, and then, cells were fixed with 4% paraformaldehyde and stained with α‐Tubulin (red) and DAPI (blue). Parasite growth in IRF3 deprived ARPE‐19 cell was measured by confocal microscopy. The number of parasites per vacuole and the number of infected cells were counted and covered to percentage. Data are representative of three independent experiments

To assess the capability of *T*.* gondii* to activate host TBK1/IRF3 signalling pathway, ARPE‐19 cells were infected with live *T*.* gondii* or treated with *Tg*ESA for 24 h, and then, Western blot analyses were performed to observe the responsiveness of the cell through measuring the phosphorylation level change of TBK1 and IRF3. A poly (dA:dT)‐treated group was used as a positive control for TBK1/IRF3 signalling pathway activation. As a result, similar with the positive control group stimulated with poly (dA:dT), *T*.* gondii* or *Tg*ESA also induced dramatic phosphorylation of TBK1 and IRF3 (Figure 1A).

Next, in order to monitor the downstream activation of IRF3 activity, IRF3 nuclear import and expression of target genes were observed by laser confocal microscopy and by RT‐PCR, respectively. As expected, *T*.* gondii* infection and *Tg*ESA treatment induced the nuclear import of IRF3 compared to unstimulated control cell (Figure 1B), and increased the downstream gene expressions, such as IFN‐β, ISG15 and ISG56 (Figure 1C). Amlexanox, an inhibitor of TBK1, clearly suppressed the *T*.* gondii*‐induced IRF3 nuclear import, indicating that this event is under control of TBK1 (Figure 1D).

To investigate the role of TBK1/IRF3 signalling pathway on *T*.* gondii* infection and growth, *T*.* gondii* infection and replication rate were measured under microscopy. As shown in Figure 1E, IRF3 deficiency had no effect on *T*.* gondii* infection rate, but clearly reduced the *T*.* gondii* replication that number of tachyzoites in each parasitophorous vacuole (PV) was mostly one or two, even after 24 h infection. Control group with normal intracellular IRF3 level showed commonly 8 tachyzoites in each PV (Figure 1E). These results suggest that *T*.* gondii* infection stimulate host IRF3 activation and translocalization through TBK1 activation and this event is indispensable for *T*.* gondii* growth in the host.

### 3.2 PI3K/AKT signalling pathway is involved in *T*.* gondii* infection‐induced IRF3 translocation but not phosphorylation

**FIGURE 2:**
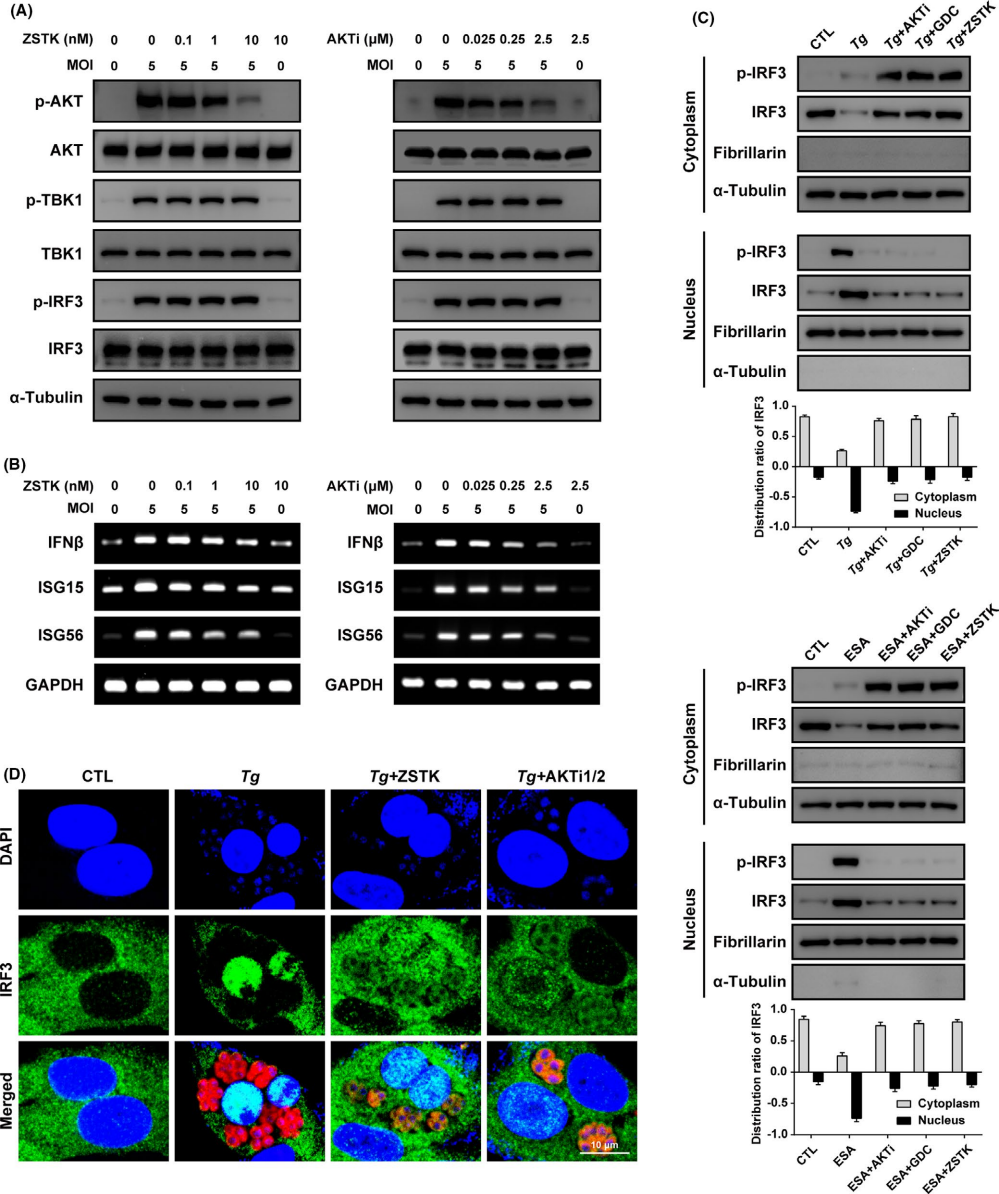
PI3K/AKT signalling is involved in *Toxoplasma gondii* infection‐induced IRF3 translocation but not phosphorylation. Followed by 4 h serum deprivation, ARPE‐19 cells pretreated with increased concentrations of PI3K inhibitor ZSTK 474 (ZSTK) and AKT inhibitor AKTi1/2 (AKTi) for 2 h and then infected with *T*.* gondii* at MOI5 for 24 h. (A) The immunoblotting was performed to detect the indicated proteins, including p‐AKT, p‐TBK1, p‐IRF3, and ATK, TBK1, IRF3. α‐Tubulin was used as the loading control. (B) The mRNA expression levels of IRF3 target genes, IFNβ and interferon‐stimulated genes (ISG15 and ISG56) were measured by RT‐PCR. (C) ARPE‐19 cells were challenged by *T*.* gondii* at MOI5 or by ESA (1 μg/ml) with pretreatment of AKT inhibitor AKTI1/2 (*Tg* + AKTi/ESA + AKTi) or PI3K inhibitors GDC‐0941 (*Tg* + GDC/ESA + GDC); ZSTK 474 (*Tg* + ZSTK/ESA + ZSTK) or not (*Tg*/ESA), or unchallenged (CTL). The nucleus and cytoplasm fractions from the cells were extracted and using Western blot analysis to examine the distribution of phosphor‐IRF3 and total IRF3. Fibrillarin and α‐tubulin were used as the nucleus and cytosol marker, respectively. (D) ARPE‐19 cells cultured in 12‐well plates were infected by RFP‐RH at MOI5 for 24 h with pretreatment of PI3K inhibitor ZSTK 474 (*Tg* + ZSTK), AKT inhibitor AKTi1/2 (*Tg* + AKTi) or not (*Tg*), or uninfected (CTL). The effect of PI3K/AKT signalling activity on IRF3 distribution was measured by Immunofluorescence. Data are representative of three independent experiments with similar results

To investigate whether PI3K/AKT signalling is involved in *T*.* gondii*‐triggered TBK1/IRF3 activity and in its downstream ISGs induction, we measured the TBK1/IRF3 phosphorylation by Western blot and ISGs mRNA levels by RT‐PCR after *T*.* gondii* infection in PI3K/AKT inhibitor pretreated ARPE‐19 cells. As shown in the results, neither PI3K nor AKT inhibitors had effect on *T*.* gondii*‐induced phosphorylation of TBK1/IRF3 (Figure 2A). Surprisingly, *T*.* gondii*‐induced production of ISGs was dramatically decreased in accordance with the increasing concentrations of PI3K/AKT inhibitors (Figure 2B). This observation suggests that PI3K/AKT signalling is not involved in the phosphorylation of TBK1/IRF3, but plays a role in IRF3 transcriptional activity.

It is well known that IRF3 is constitutively expressed and localized in the cytoplasm in a latent form. But, in response to the pathogens infection, IRF3 can be activated by phosphorylation of the C‐terminal region at seven Ser/Thr residues (Ser385, Ser386, Ser396, Ser398, Ser402, Ser405 and Thr404). Phosphorylated IRF3 shuttles into the nucleus, where they initiate transcription of target genes.^12^ Therefore, the Western blot analysis with fractionated cell extracts was performed to investigate whether PI3K/AKT is involved in the translocation process or not. ARPE‐19 cells were pretreated with PI3K‐specific inhibitors, GDC‐0941/ZSTK474, and AKT specific inhibitor AKTi1/2 for 2 h, and followed by challenging with *T*.* gondii* (RFP‐RH) or *Tg*ESA. As shown in Figure 2C, *T*. *gondii* infection or *Tg*ESA treatment induced the host IRF3 phosphorylation and moved the IRF3 from the cytosol to the nucleus, but inhibition of PI3K/AKT pathway clearly nullify the effect that even with its phosphorylation, host IRF3 stayed in the cytosol fraction. Immunofluorescence images also confirmed that *T*.* gondii* infection induced IRF3 nuclear import was dramatically inhibited with PI3K/AKT inhibitors that most of IRF3 was accumulated in the host cytoplasm even with the parasite infection (Figure 2D).

These data suggested that, PI3K/AKT signalling pathway is involved in the transcriptional activation of IRF3 by promoting IRF3 nuclear translocation in response to *T*.* gondii* infection in ARPE‐19 cells.

### 3.3 FAF1 expression is reduced in the host by *T*.* gondii* infection

Fas‐associated factor 1 (FAF1) is a Fas‐binding pro‐apoptotic protein that is a component of the death‐inducing signalling complex in Fas‐mediated apoptosis.^27^ It has also been demonstrated that FAF1 is involved in the immune response by physically interacts with NF‐κB p65 to prevent its translocation into the nucleus and downregulate the target genes.^28^ Thus, the change of host FAF1 protein and mRNA levels after *T*.* gondii* stimulation were measured to investigate whether FAF1 is associated with the process of parasite infection.

**FIGURE 3:**
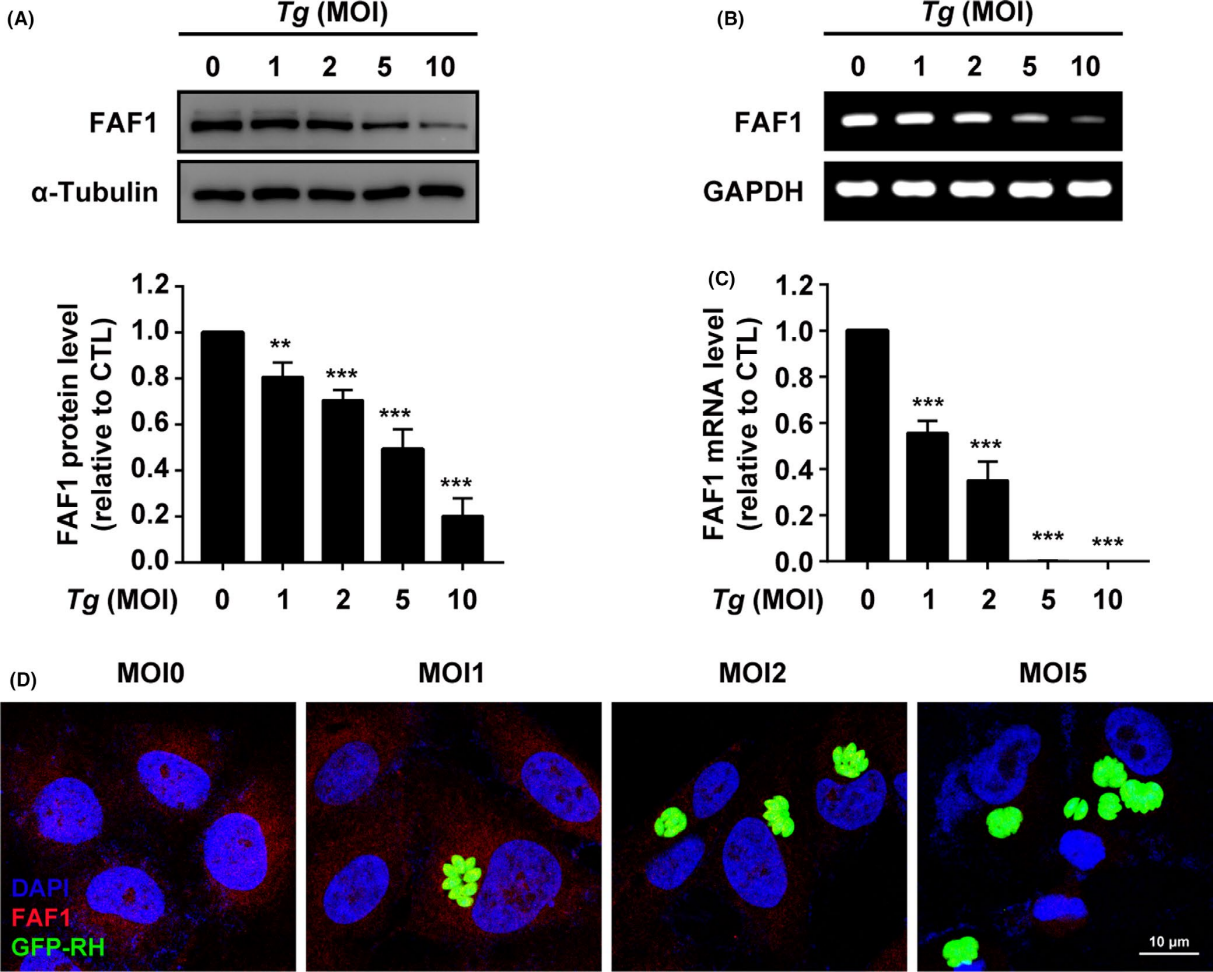
*Toxoplasma gondii* infection caused a reduction of FAF1. (A) FAF1 protein level was measured by Western blot analysis. (B) The mRNA expression levels of FAF1 were measured by RT‐PCR. (C) and Real‐time PCR. (D) ARPE‐19 cells infected with *T*.* gondii* were stained with FAF1 antibody, the levels of FAF1 were analysed by confocal microscopy. Similar results were obtained in three independent experiments. **, *p* < 0.01; ***, *p* < 0.001, as compared to the MOI 0 group

Western blot analysis showed that FAF1 expression decreased in a MOI‐dependent manner upon *T*.* gondii* tachyzoite infection, and, at MOI 10, host intracellular FAF1 level reached its most minimal level (Figure 3A). To confirm that endogenous FAF1 synthesis was downregulated by *T*.* gondii*, FAF1 mRNA expression level was measured using RT‐PCR (Figure 3B) and quantified by qPCR (Figure 3C). Indeed, FAF1 mRNA level was dramatically diminished in accordance with the increasing MOI of *T*.* gondii* (Figure 3B,C). Coincidently, the measurement of intracellular FAF1 level by fluorescent microscopic also supported our hypothesis that *T*.* gondii* infection lead to a MOI‐dependent downregulation of FAF1 expression in the host cell (Figure 3D).

### 3.4 *T*.* gondii* infection‐induced downregulation of FAF1 is dependent on PI3K/AKT/FOXO1 pathway

**FIGURE 4:**
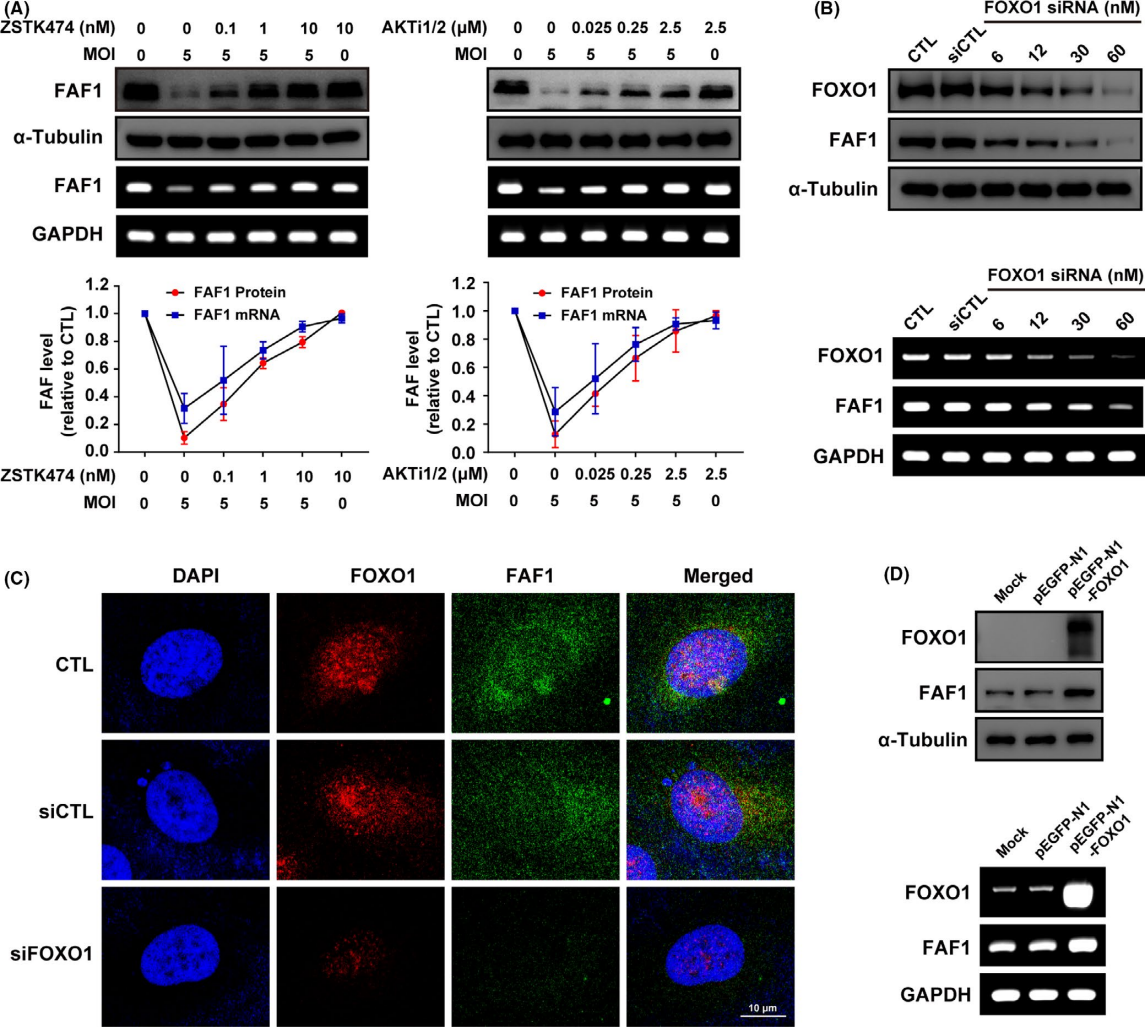
*Toxoplasma gondii*‐induced downregulation of FAF1 is dependent on PI3K/AKT‐mediated FOXO1. (A) ARPE‐19 cells were pretreated with PI3K inhibitor ZSTK474 or AKT inhibitor AKTi1/2 at the indicated dosage for 2 h and then infected with *T*.* gondii* at MOI5 for 24 h. The protein level of FAF1 was measured by Western blot analysis, and the mRNA expression level was measured by RT‐PCR. (B) ARPE‐19 cells were transiently transfected with either different concentrations of FOXO1 siRNA (siFOXO1) or control scrambled siRNA (siCTL) or untransfected (CTL). The protein and mRNA expression levels of FOXO1 and FAF1 were measured by Western blot and RT‐PCR, respectively. (C) The levels of FOXO1 and FAF1 were analysed by fluorescent microscopy. (D) ARPE‐19 cells were transiently transfected with either pEGFP‐N1 or pEGFP‐N1‐FOXO1 or untransfected (Mock). The protein and mRNA expression levels of FOXO1 and FAF1 were measured by Western blot and RT‐PCR, respectively. All data shown are representative of three independent experiments with similar results

To identify the control mechanism for the reduction of FAF1 induced by *T*.* gondii*, we tested the involvement of PI3K/AKT signalling pathway on this event based on its effect on IRF3 translocalization. ARPE‐19 cells were pretreated with PI3K inhibitor ZSTK474 or AKT inhibitor AKTi1/2 at the designate concentration for 2 h and then stimulated with *T*.* gondii* for 24 h. Indeed, it was found that the reduction of FAF1 level induced by *T*.* gondii* was reversed by inhibition of PI3K/AKT signalling pathway (Figure 4A). Based on the result, we confirmed that involvement of PI3K/AKT pathway in FAF1 regulation, and then, we suspected that FOXO1, a well‐known downstream transcription factor regulated by AKT, is a potential transcription factor for regulating FAF1 gene expression in ARPE‐19 cells.

To evaluate this possibility, we adopted gene silencing technique to monitor the effect of FOXO1 deficiency on host FAF1 level. ARPE‐19 cells were transfected with FOXO1 siRNA, and the FAF1 protein level was analysed by Western blot technique (Figure 4B upper). The outcome showed that depletion the endogenous FOXO1 by siRNA markedly diminished host FAF1 protein level. Following RT‐PCR, examination supported the idea that FAF1 protein reduction by FOXO1 is originated from downregulation FAF1 mRNA transcription in ARPE‐19 cells (Figure 4B lower).

Consistently, confocal microscopy images for endogenous FAF1 also illustrated that siRNA‐mediated FOXO1 gene silencing caused a dramatic reduction of FAF1 compared to the control siRNA‐treated group (Figure 4C), whereas FOXO1 overexpression indeed enhanced the host FAF1 levels (Figure 4D).

### 3.5 FOXO1‐mediated FAF1 expression modulates IRF3 nuclear translocation, downstream gene expression and infected *T*.* gondii* growth

**FIGURE 5:**
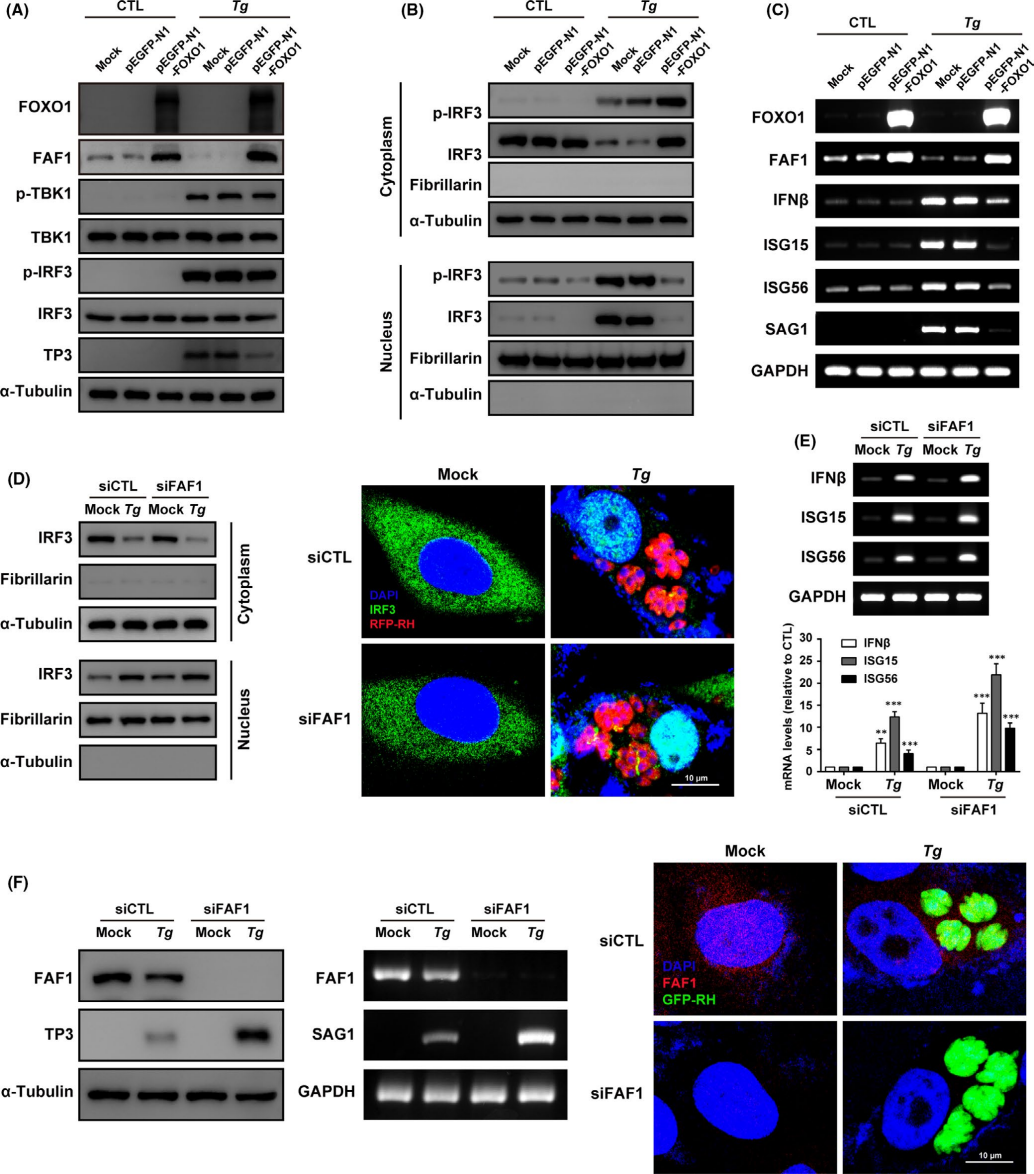
FAF1 functions as negative regulator for IRF3 nuclear translocation and prevents infected *Toxoplasma gondii* growth. ARPE‐19 cells were transiently transfected with either pEGFP‐N1 or pEGFP‐N1‐FOXO1 or untransfected (Mock), and then infected with *T*.* gondii* at MOI5 (*Tg*) or not (CTL). (A) FOXO1 overexpression mediated FAF1 upregulation has no effect on the phosphorylation level of TBK1 and IRF3. (B) FAF1 upregulation by FOXO1 overexpression suppressed *T*.* gondii*‐induced IRF3 nuclear import. (C) FOXO1 overexpression mediated FAF1 upregulation inhibited *T*.* gondii*‐induced production of IRF3 target genes. ARPE‐19 cells were transiently transfected with either FAF1 siRNA (siFAF1) or control scrambled siRNA (siCTL) and then infected with *T*.* gondii* at MOI5 (*Tg*) or not (Mock). (D) The nuclear translocation of IRF3 was monitored by two independent techniques: Western blot and Confocal microscopy. (E) The mRNA levels of IRF3 target genes were measured by RT‐PCR. (F) Whole‐cell proteins were analysed by immunoblotting to detect FAF1 and *T*.* gondii* TP3 proteins. Parasite growth was also measured by RT‐PCR using the SAG1 primer, and confocal microscope with *T*.* gondii* tachyzoites GFP‐RH. Similar results were obtained in three independent experiments. **, *p* < 0.01; ***, *p* < 0.001, as compared to the control group

Next, we examined whether FAF1 is involved in IRF3 nuclear translocation induced by *T*.* gondii*. FOXO1 overexpression‐dependent FAF1 upregulation had no effect on the phosphorylation of TBK1/IRF3 by *T*.* gondii* infection (Figure 5A), but the IRF3 nuclear import was dramatically suppressed by the condition (Figure 5B). Accordingly, the production levels of IRF3 downstream ISGs were also obviously inhibited, even in the presence of *T*.* gondii* infection (Figure 5C). Silencing FAF1 gene revealed opposite results that IRF3 nuclear import (Figure 5D left) and downstream ISGs expression were enhanced compare to control groups (Figure 5E). This observation was also confirmed with confocal microscopy of IRF3 in the FAF1‐knocked‐down cells infected with *T*.* gondii* (RFP‐RH) (Figure 5D right).

Based on the evidences, the impact of host FAF1 level on *T*.* gondii* growth in ARPE‐19 cells was investigated. The host cell with FOXO1 overexpression, followed by the increase of FAF1 protein, resulted in notable reduction of the SAG1 protein (TP3, SAG1 gene product, *T*.* gondii* surface antigen) (Figure 5A) and its mRNA (Figure 5C), which suggests the effective inhibition of *T*.* gondii* growth. To the contrary, in the FAF1 silenced group, the SAG1 protein and mRNA level were increased, and this result indicates that host suppression mechanism for the parasite growth is impaired. Confocal microscopic image also provided the evidence that in the FAF1 deficiency, *T*.* gondii* replication in a PV is enhanced compared to the control group with normal FAF1 level (Figure 5F).

All these data indicate that FAF1 gene expression can be regulated by PI3K/AKT pathway through FOXO1 transcription factor and *T*.* gondii* can manipulate this event on purpose. This process of *T*.* gondii* to snatch and activate host specific signalling pathway may be essential in *T*.* gondii* survival and proliferation to continue parasite life in ARPE‐19 cells.

## 4 DISCUSSION

Innate immune system, which presents in all cell types, plays a critical role in defending the host from various infectious agents, such as virus, bacteria and even. In response to the infection, the host pathogen recognition receptors (PRRs) recognize the specific agent or the antigen and evoke the production of type I IFNs including IFN‐α and IFN‐β. Type I IFNs promote an anti‐infection state by transcriptional induction of approximately 300 IFN‐stimulated genes (ISGs) in an autocrine or paracrine manners.^29^, ^30^ The previous study shown that during the test of the antiviral effects of more than 350 individual human ISGs, 25 genes were revealed to enhance certain virus infectivity.^31^ These genes expression is highly dependent on activity of TBK1/IRF3 signalling.

Interferon regulatory factor 3 (IRF3) has been known as a transcription regulator of many cellular responses in many cell types that is known to be critical for innate immunity against viral infection. Activation of IRF3 requires phosphorylation on specific serine residues by the kinase TANK‐binding kinase 1 (TBK1), which leads to the translocation of IRF3 into the nucleus. Although TBK1‐IRF3 signal axis had its reputation for the host defence mechanism, it also has been reported that the activation of host IRF3 is also indispensable for the replication of the protozoan parasite, *T*. *gondii*.^13^, ^14^ This requirement is also observed and confirmed in our *T*.* gondii* infection model that the TBK1 inactivation by specific inhibitor Amlexanox or IRF3 depletion by gene silencing dramatically restrained the parasite proliferation. These observations suggest that the activation of host TBK1/IRF3 signalling pathway is also essential for development of ocular toxoplasmosis.

Interestingly, *T*.* gondii* has been known for taking advantage of specific host signalling pathways, such as PI3K/AKT pathway, for changing host environment and for supporting its growth. More than a decade ago, there was evidence that *T*.* gondii* exploits host Gi‐dependent PI3K/AKT signalling to maintain host cell survival and parasite persistence.^15^ *Toxoplasma gondii*‐mediated EGFR‐dependent AKT activation also has been linked to inhibition of autophagy of *T*.* gondii*‐infected cells and raised the possibility that AKT signalling as a pathway critical for the pathogen survival.^32^ *Toxoplasma gondii* also has evolved a unique mechanism to suppress the host ROS generation by reducing NOX4, which expression is regulated by PI3K/AKT signalling.^16^, ^33^ The findings of these multiple host signal activations by *T*.* gondii* raised a question for the presence of crosslink or hierarchy between the signals.

It has been reported that PI3K/AKT pathway functions as upstream of IRF3 activation in the type I IFN‐producing pathway since AKT inhibitors abrogates IRF3 phosphorylation in *SAMHD*‐deficient THP‐1 cells.^34^ There is another report showing that IRF3 activation induced by Sendai virus infection was attenuated by PI3K inhibitor or AKT dominant‐negative mutant in BMDM.^35^ Therefore, we hypothesized that *T*.* gondii* may take advantage of this interaction between PI3K/AKT signalling and TBK1/IRF3 pathway while its infection period. Surprisingly, we observed that PI3K/AKT inhibitors prevented IRF3 from nuclear translocation and downstream gene expression after *T*.* gondii* infection, even with its phosphorylation intact in ARPE‐19 cells. These findings suggest that IRF3 downstream gene expression is dependent on two processes, IRF3 phosphorylation and IRF3 nuclear import mechanism, which is under control of PI3K/AKT activity.

Among the numerous possible control mechanisms for nuclear translocation of transcription factor, we focused on Fas‐associated factor 1 (FAF1) as our player because of their known properties to bind with transcription factors. Originally, FAF1 has been known as a Fas‐binding pro‐apoptotic protein that is a component of the death‐inducing signalling complex in Fas‐mediated apoptosis.^22^ Yet, recently, involvement of FAF1 in the immune response has been demonstrated. As we mentioned, physical interaction of FAF1 with NF‐ĸB p65 prevents translocation of NF‐kB into the nucleus and downregulate the target genes.^28^ Information about the interaction between FAF1 and TBK1 signalling pathway is rather limited, but there is a report that FAF1 can inhibit poly(I:C)‐ and respiratory syncytial virus (RSV)‐induced production of type I interferons (IFNs) by blocking the physically interaction between IRF3 and IPO5/importin‐β3 and, thereby, IRF3 translocating into the nucleus.^25^

Several mechanisms for FAF1 activity regulation have been proposed, and among them, interestingly, was AKT. AKT can directly phosphorylates FAF1 at Ser582, which disrupts the FAF1‐VCP complex and reduces FAF1 at the plasma membrane.^36^ But in our *T*.* gondii* infection model, *T*.* gondii* infection to ARPE‐19 cells dramatically reduced host intracellular FAF1, and it was reversed by PI3K inhibition. Therefore, we rather focused on FAF1 gene expression regulation instead of posttranslational modification of FAF1. Recent study has been shown that FAF1 is one of the 46 identified targets of FOXO1, among all species (*C*. *elegans*, *Drosophila*, mouse and human).^37^ Forkhead box O (FOXO) transcription factor is a major downstream targets of PI3K/AKT signalling, and phosphorylation of FOXOs by AKT inhibits its transcriptional functions by promoting their interaction with 14‐3‐3 protein that results in nuclear exclusion, and contributes to cell survival, growth and proliferation.^17^

In silico analysis of 1 kb human *FAF1* promoter sequence (data not shown) also identified one potential Forkhead Response Element (FRE), the consensus FOXO1 transcription factors binding sequence (GAAAACA).^38^ The presence of this sequence in the promoter of FAF1 implies that FOXO1 may have a function as a transcription factor for FAF1. Indeed, there is a high probability that FAF1 expression level can be regulated by FOXO1 since FOXO1 deficiency dramatically diminished FAF1 mRNA level, while FOXO1 overexpression elevated it. Our data also showed that FAF1 depletion facilitate IRF3 nuclear import followed by enhancement of *T*.* gondii* replication, suggesting that FAF1 plays a key role in regulating the innate immune system by inhibiting the translocation of IRF3 into the nucleus and preventing *T*.* gondii* survival.

The role of ISGs for *T*.* gondii* growth and survival is not fully understood yet. ISG15 has been reported to be detrimental to the parasite by linking the autophagy‐mediated control to ubiquitination of the vacuole.^38^, ^39^ Yet, here we show that, in ISG15 deficient condition, *T*.* gondii* replication was suppressed, while, in ISG15 overexpress condition, *T*.* gondii* proliferation was significantly enhanced (Figure S1). The results indicated that ISG15 is essential for *T*.* gondii* growth in ARPE‐19 cells. As IRF3 is a target for ISGylation, conjugation of ISG15 positively regulates IRF3 by preventing IRF3 ubiquitination and subsequent degradation. In addition, we also have data shown that the protease inhibitor MG‐132 restored ISG15 deficiency‐induced suppression of *T*.* gondii* growth. We presume that ISG15 contributes to *T*.* gondii* proliferation by preventing IRF3 from degradation. ISG56, is reported as a mediator of negative‐feedback regulation of virus‐triggered induction by type I IFNs and cellular antiviral responses.^29^ However, Majumdar and his colleagues have shown that ISG56 is capable of promoting *T*.* gondii* replication, in cell cultures or in mice.^13^ We also provide the supporting results that silencing of ISG56 in the host resulted in defect of *T*.* gondii* proliferation, significantly (Figure S2), which means that it is also indispensable for *T*.* gondii* in ARPE‐19 cells. These findings suggest that *T*.* gondii* has more complicated usage of host signal modifiers and further studies would be necessary to decode the antigen or host stimulating factors which would determine the fate of host and parasite.

**FIGURE 6:**
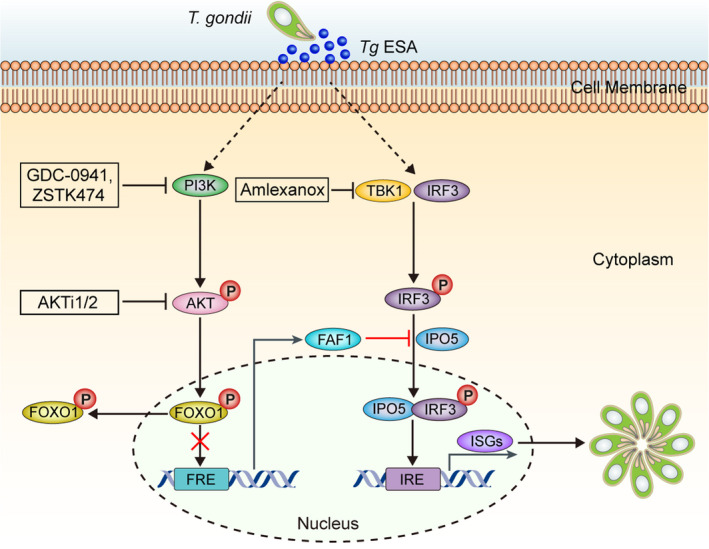
Schematic model of downregulation of FAF1 by *Toxoplasma gondii* to promote IRF3 nuclear translocation

Collectively, our results show that PI3K/AKT pathway is an indispensable component for controlling of host IRF3 nuclear import on response to *T*.* gondii* infection, and FAF1 is an essential element for connection and efficient activation the host TBK1‐IRF3 signal axis, which contribute to the survival and growth of *T*.* gondii* (Figure 6). These findings suggest that PI3K/AKT and FAF1 could be useful therapeutic targets to manipulate TBK1‐IRF3 relied immune responses and to improve parasite control against *T*.* gondii* infection.

## CONFLICT OF INTEREST

No potential conflicts of interest were disclosed.

## AUTHOR CONTRIBUTION

**Fei‐Fei Gao:** Conceptualization (lead); Data curation (lead); Formal analysis (equal); Investigation (equal); Methodology (lead); Project administration (lead); Resources (equal); Software (equal); Validation (equal); Writing‐original draft (supporting). **Juan‐Hua Quan:** Conceptualization (lead); Data curation (equal); Formal analysis (lead); Funding acquisition (equal); Investigation (equal); Methodology (lead); Project administration (equal); Resources (equal); Software (lead); Supervision (equal); Visualization (lead); Writing‐original draft (supporting). **In‐Wook Choi:** Methodology (equal); Project administration (equal); Resources (equal); Validation (equal). **Yeon‐Jae Lee:** Methodology (equal); Project administration (equal); Resources (equal). **Seul‐Gi Jang:** Methodology (equal); Project administration (equal); Resources (equal). **Jae‐Min Yuk:** Conceptualization (equal); Data curation (equal); Formal analysis (equal); Investigation (equal); Supervision (equal). **Young‐Ha Lee:** Conceptualization (lead); Data curation (equal); Formal analysis (equal); Funding acquisition (equal); Resources (equal); Supervision (lead); Validation (lead); Visualization (lead); Writing‐original draft (lead); Writing‐review & editing (lead). **Guang‐Ho Cha:** Conceptualization (lead); Formal analysis (lead); Funding acquisition (lead); Project administration (lead); Resources (lead); Supervision (lead); Validation (lead); Visualization (lead); Writing‐original draft (lead); Writing‐review & editing (lead).

## Supporting information

Figure S1

Figure S2

Supplementary Material

## Data Availability

The data that support the findings of this study are available on request from the corresponding author. The data are not publicly available due to privacy or ethical restrictions.

## References

[1] Montoya JG , Liesenfeld O . Toxoplasmosis. Lancet. 2004;363(9425):1965‐1976. 15194258 10.1016/S0140-6736(04)16412-X

[2] Vallochi AL , Nakamura MV , Schlesinger D , et al. Ocular toxoplasmosis: more than just what meets the eye. Scand J Immunol. 2002;55(4):324‐328. 11967112 10.1046/j.1365-3083.2002.01052.x

[3] Li W , Nagineni CN , Hooks JJ , Chepelinsky AB , Egwuagu CE . Interferon‐gamma signaling in human retinal pigment epithelial cells mediated by STAT1, ICSBP, and IRF‐1 transcription factors. Invest Ophthalmol Vis Sci. 1999;40(5):976‐982. 10102295

[4] Nagineni CN , Detrick B , Hooks JJ . Transforming growth factor‐beta expression in human retinal pigment epithelial cells is enhanced by *Toxoplasma* *gondii*: a possible role in the immunopathogenesis of retinochoroiditis. Clin Exp Immunol. 2002;128(2):372‐378. 11985530 10.1046/j.1365-2249.2002.01815.x PMC1906397

[5] Choi SH , Park SJ , Cha G‐H , et al. *Toxoplasma* *gondii* protects against H(2)O(2) ‐induced apoptosis in ARPE‐19 cells through the transcriptional regulation of apoptotic elements and downregulation of the p38 MAPK pathway. Acta Ophthalmol. 2011;89(4):e350‐e356. 21385331 10.1111/j.1755-3768.2011.02113.x

[6] Nogueira AR , Leve F , Morgado‐Diaz J , Tedesco RC , Pereira MC . Effect of *Toxoplasma* *gondii* infection on the junctional complex of retinal pigment epithelial cells. Parasitology. 2016;143(5):568‐575. 26928468 10.1017/S0031182015001973

[7] Song HB , Jun H‐O , Kim JH , Lee Y‐H , Choi M‐H , Kim JH . Disruption of outer blood‐retinal barrier by *Toxoplasma* *gondii*‐infected monocytes is mediated by paracrinely activated FAK signaling. PLoS One. 2017;12(4):e0175159. 28406972 10.1371/journal.pone.0175159 PMC5390985

[8] Tamura T , Yanai H , Savitsky D , Taniguchi T . The IRF family transcription factors in immunity and oncogenesis. Annu Rev Immunol. 2008;26:535‐584. 18303999 10.1146/annurev.immunol.26.021607.090400

[9] Sato M , Suemori H , Hata N , et al. Distinct and essential roles of transcription factors IRF‐3 and IRF‐7 in response to viruses for IFN‐alpha/beta gene induction. Immunity. 2000;13(4):539‐548. 11070172 10.1016/s1074-7613(00)00053-4

[10] Lin R , Heylbroeck C , Pitha PM , Hiscott J . Virus‐dependent phosphorylation of the IRF‐3 transcription factor regulates nuclear translocation, transactivation potential, and proteasome‐mediated degradation. Mol Cell Biol. 1998;18(5):2986‐2996. 9566918 10.1128/mcb.18.5.2986 PMC110678

[11] Servant MJ , ten Oever B , LePage C , et al. Identification of distinct signaling pathways leading to the phosphorylation of interferon regulatory factor 3. J Biol Chem. 2001;276(1):355‐363. 11035028 10.1074/jbc.M007790200

[12] Servant MJ , Grandvaux N , tenOever BR , Duguay D , Lin R , Hiscott J . Identification of the minimal phosphoacceptor site required for in vivo activation of interferon regulatory factor 3 in response to virus and double‐stranded RNA. J Biol Chem. 2003;278(11):9441‐9447. 12524442 10.1074/jbc.M209851200

[13] Majumdar T , Chattopadhyay S , Ozhegov E , et al. Induction of interferon‐stimulated genes by IRF3 promotes replication of *Toxoplasma* *gondii* . PLoS Pathog. 2015;11(3):e1004779. 25811886 10.1371/journal.ppat.1004779 PMC4374777

[14] Majumdar T , Sharma S , Kumar M , et al. Tryptophan‐kynurenine pathway attenuates beta‐catenin‐dependent pro‐parasitic role of STING‐TICAM2‐IRF3‐IDO1 signalosome in *Toxoplasma* *gondii* infection. Cell Death Dis. 2019;10(3):161. 30770800 10.1038/s41419-019-1420-9 PMC6377608

[15] Kim L , Denkers EY . *Toxoplasma* *gondii* triggers Gi‐dependent PI 3‐kinase signaling required for inhibition of host cell apoptosis. J Cell Sci. 2006;119(Pt 10):2119‐2126. 16638808 10.1242/jcs.02934

[16] Zhou W , Quan J‐H , Lee Y‐H , Shin D‐W , Cha G‐H . *Toxoplasma* *gondii* proliferation require down‐regulation of host Nox4 expression via activation of PI3 kinase/Akt signaling pathway. PLoS One. 2013;8(6):e66306. 23824914 10.1371/journal.pone.0066306 PMC3688893

[17] Tzivion G , Dobson M , Ramakrishnan G . FoxO transcription factors; Regulation by AKT and 14‐3‐3 proteins. Biochim Biophys Acta. 2011;1813(11):1938‐1945. 21708191 10.1016/j.bbamcr.2011.06.002

[18] Dejean AS , Hedrick SM , Kerdiles YM . Highly specialized role of Forkhead box O transcription factors in the immune system. Antioxid Redox Signal. 2011;14(4):663‐674. 20673126 10.1089/ars.2010.3414 PMC3021368

[19] Cabrera‐Ortega AA , Feinberg D , Liang Y , Rossa C , Graves DT . The role of forkhead box 1 (FOXO1) in the immune system: dendritic cells, T cells, B cells, and hematopoietic stem cells. Crit Rev Immunol. 2017;37(1):1‐14. 29431075 10.1615/CritRevImmunol.2017019636 PMC6085137

[20] Graves DT , Milovanova TN . Mucosal immunity and the FOXO1 transcription factors. Front Immunol. 2019;10:2530. 31849924 10.3389/fimmu.2019.02530 PMC6896163

[21] Litvak V , Ratushny AV , Lampano AE , et al. A FOXO3‐IRF7 gene regulatory circuit limits inflammatory sequelae of antiviral responses. Nature. 2012;490(7420):421‐425. 22982991 10.1038/nature11428 PMC3556990

[22] Chu K , Niu X , Williams LT . A Fas‐associated protein factor, FAF1, potentiates Fas‐mediated apoptosis. Proc Natl Acad Sci USA. 1995;92(25):11894‐11898. 8524870 10.1073/pnas.92.25.11894 PMC40509

[23] Menges CW , Altomare DA , Testa JR . FAS‐associated factor 1 (FAF1): diverse functions and implications for oncogenesis. Cell Cycle. 2009;8(16):2528‐2534. 19597341 10.4161/cc.8.16.9280 PMC2739729

[24] Kim TH , Lee H‐C , Kim J‐H , et al. Fas‐associated factor 1 mediates NADPH oxidase‐induced reactive oxygen species production and proinflammatory responses in macrophages against Listeria infection. PLoS Pathog. 2019;15(8):e1008004. 31412082 10.1371/journal.ppat.1008004 PMC6709923

[25] Song S , Lee J‐J , Kim H‐J , Lee JY , Chang J , Lee K‐J . Fas‐associated factor 1 negatively regulates the antiviral immune response by inhibiting translocation of interferon regulatory factor 3 to the nucleus. Mol Cell Biol. 2016;36(7):1136‐1151. 26811330 10.1128/MCB.00744-15 PMC4800795

[26] Son ES , Nam HW . Detection and characterization of excretory/secretory proteins from *Toxoplasma* *gondii* by monoclonal antibodies. Korean J Parasitol. 2001;39(1):49‐56. 11301590 10.3347/kjp.2001.39.1.49 PMC2721065

[27] Ryu SW , Lee S‐J , Park M‐Y , Jun J‐I , Jung Y‐K , Kim E . Fas‐associated factor 1, FAF1, is a member of Fas death‐inducing signaling complex. J Biol Chem. 2003;278(26):24003‐24010. 12702723 10.1074/jbc.M302200200

[28] Park MY , Jang HD , Lee SY , Lee KJ , Kim E . Fas‐associated factor‐1 inhibits nuclear factor‐kappaB (NF‐kappaB) activity by interfering with nuclear translocation of the RelA (p65) subunit of NF‐kappaB. J Biol Chem. 2004;279(4):2544‐2549. 14600157 10.1074/jbc.M304565200

[29] Li Y , Li C , Xue P , et al. ISG56 is a negative‐feedback regulator of virus‐triggered signaling and cellular antiviral response. Proc Natl Acad Sci USA. 2009;106(19):7945‐7950. 19416887 10.1073/pnas.0900818106 PMC2683125

[30] Xu L , Wang W , Li Y , et al. RIG‐I is a key antiviral interferon‐stimulated gene against hepatitis E virus regardless of interferon production. Hepatology. 2017;65(6):1823‐1839. 28195391 10.1002/hep.29105

[31] Schoggins JW , MacDuff DA , Imanaka N , et al. Pan‐viral specificity of IFN‐induced genes reveals new roles for cGAS in innate immunity. Nature. 2014;505(7485):691‐695. 24284630 10.1038/nature12862 PMC4077721

[32] Muniz‐Feliciano L , Van Grol J , Portillo J‐A , et al. *Toxoplasma* *gondii*‐induced activation of EGFR prevents autophagy protein‐mediated killing of the parasite. PLoS Pathog. 2013;9(12):e1003809. 24367261 10.1371/journal.ppat.1003809 PMC3868508

[33] Choi HG , Gao F‐F , Zhou W , et al. The role of PI3K/AKT pathway and NADPH oxidase 4 in host ROS manipulation by *Toxoplasma* *gondii* . Korean J Parasitol. 2020;58(3):237‐247. 32615737 10.3347/kjp.2020.58.3.237 PMC7338895

[34] Oh C , Ryoo J , Park K , et al. A central role for PI3K‐AKT signaling pathway in linking SAMHD1‐deficiency to the type I interferon signature. Sci Rep. 2018;8(1):84. 29311560 10.1038/s41598-017-18308-8 PMC5758801

[35] Yeon SH , Song MJ , Kang H‐R , Lee JY . Phosphatidylinositol‐3‐kinase and Akt are required for RIG‐I‐mediated anti‐viral signalling through cross‐talk with IPS‐1. Immunology. 2015;144(2):312‐320. 25158146 10.1111/imm.12373 PMC4298425

[36] Xie F , Jin K , Shao L , et al. FAF1 phosphorylation by AKT accumulates TGF‐beta type II receptor and drives breast cancer metastasis. Nat Commun. 2017;8:15021. 28443643 10.1038/ncomms15021 PMC5414047

[37] Webb AE , Kundaje A , Brunet A . Characterization of the direct targets of FOXO transcription factors throughout evolution. Aging Cell. 2016;15(4):673‐685. 27061590 10.1111/acel.12479 PMC4933671

[38] Biggs WH 3rd , Cavenee WK , Arden KC . Identification and characterization of members of the FKHR (FOX O) subclass of winged‐helix transcription factors in the mouse. Mamm Genome. 2001;12(6):416‐425. 11353388 10.1007/s003350020002

[39] Bhushan J , Radke JB , Perng YC , et al. ISG15 connects autophagy and IFN‐gamma‐dependent control of *Toxoplasma* *gondii* infection in human cells. MBio. 2020;11(5):e00852‐20. 33024031 10.1128/mBio.00852-20 PMC7542356

