# Supplementary figures and images for: FAF1 downregulation by Toxoplasma gondii enables host IRF3 mobilization and promotes parasite growth
Source: J Cell Mol Med. 2021 Aug 31;25(19):9460–72. doi: 10.1111/jcmm.16889 (PMC8500981; doi:10.1111/jcmm.16889)

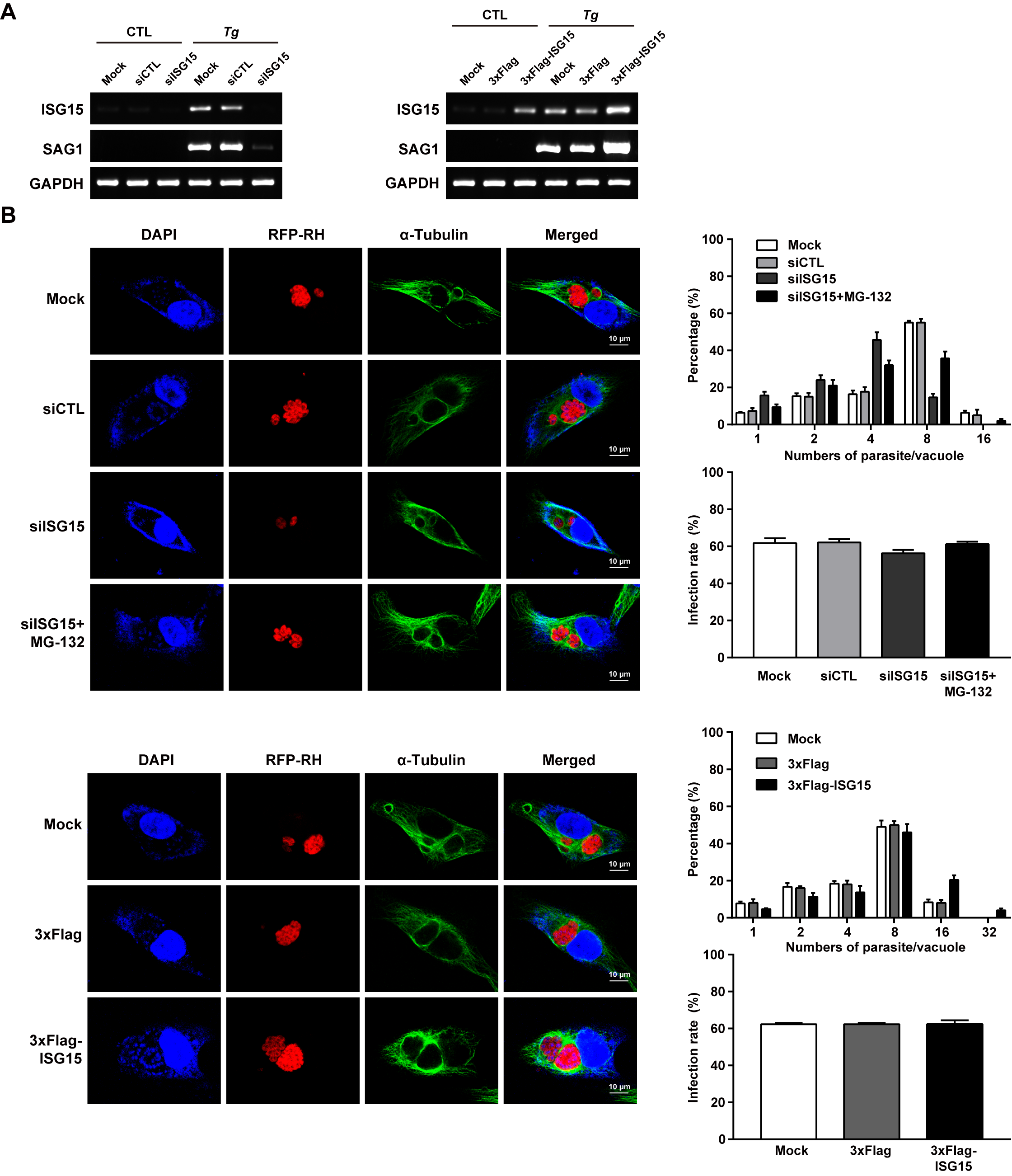

Supplement: Supplementary file 1 — Figure S1 [file JCMM-25-9460-s003.tif]

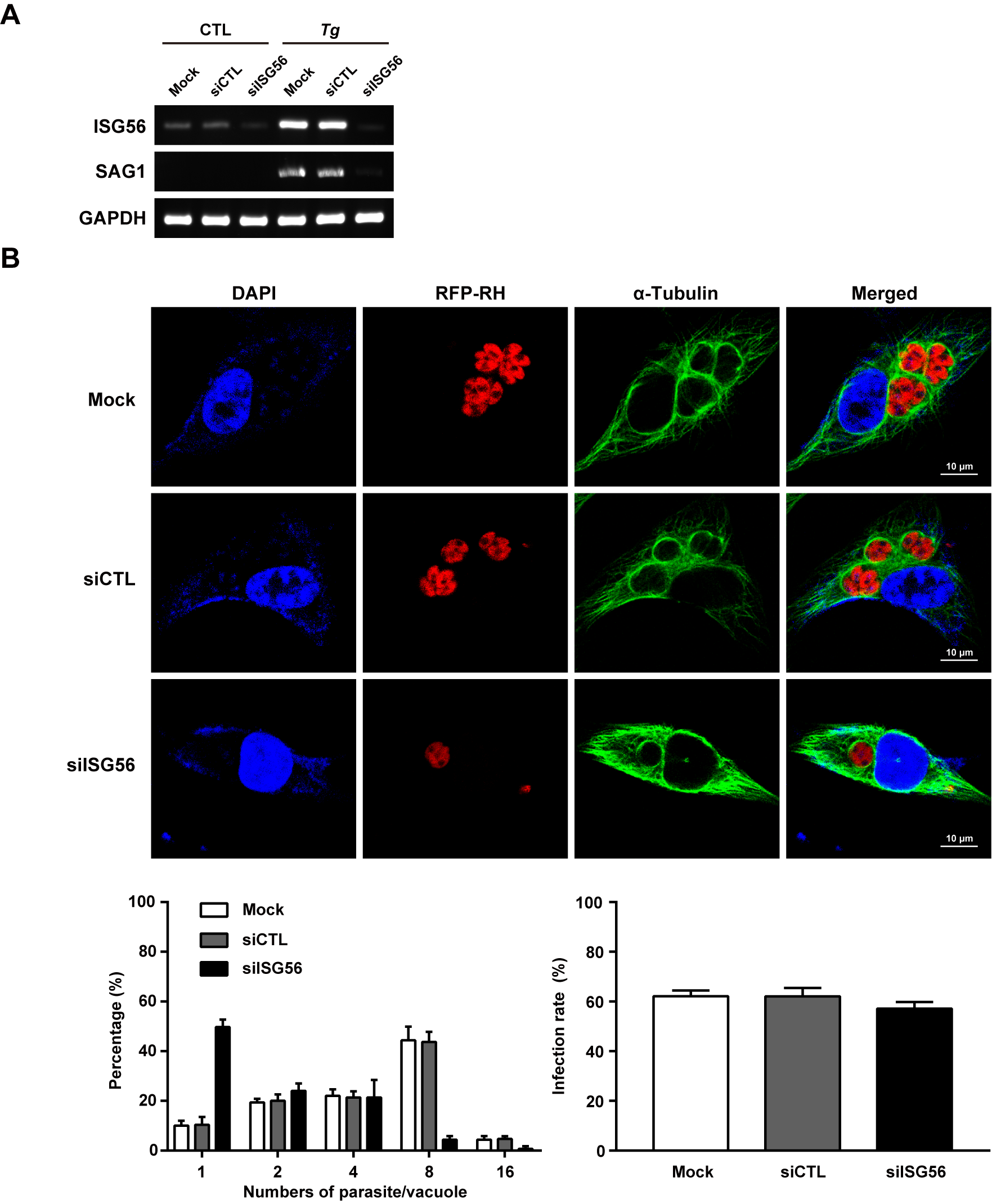

Supplement: Supplementary file 2 — Figure S2 [file JCMM-25-9460-s001.tif]
