# Supplementary material for: FAF1 downregulation by Toxoplasma gondii enables host IRF3 mobilization and promotes parasite growth
Source: J Cell Mol Med. 2021 Aug 31;25(19):9460–72. doi: 10.1111/jcmm.16889 (PMC8500981; doi:10.1111/jcmm.16889)
Supplement: Supplementary file 3 — Supplementary Material [file JCMM-25-9460-s002.docx]

**Figure S1. ISG15 is pivotal for *T. gondii* growth in ARPE-19 cells.** ARPE-19 cells were transiently transfected with control siRNA (siCTL), ISG15 siRNA (siISG15), p3x FlAG-CMV-10 expression vector (3xFlag), or p3x FlAG-CMV-10-ISG15 (3xFlag-ISG15) or untransfected (Mock), and then infected with *T. gondii* at MOI5 (*Tg*) or not (CTL) for 24 hrs. **(A)** Parasite growth was measured by RT-PCR using the SAG1 primer. **(B)** Confocal microscope with *T. gondii* tachyzoites RFP-RH. The number of parasites per vacuole and the number of infected host cells were counted and converted to percentage. Data were representative of three independent experiments.

**Figure S2. ISG56 is pivotal for *T. gondii* growth in ARPE-19 cells.** ARPE-19 cells were transiently transfected with control siRNA (siCTL) or ISG56 siRNA (siISG56), or untransfected (Mock), and then infected with *T. gondii* at MOI5 (*Tg*) or not (CTL) for 24 hrs. **(A)** Parasite growth was measured by RT-PCR using the SAG1 primer. **(B)** Confocal microscope with *T. gondii* tachyzoites RFP-RH. The number of parasites per vacuole and the number of infected host cells were counted and converted to percentage. Data were representative of three independent experiments.
